# Evaluation of the Efficacy of Mineralized Dentin Graft in the Treatment of Intraosseous Defects: An Experimental In Vivo Study

**DOI:** 10.3390/medicina58010103

**Published:** 2022-01-10

**Authors:** Nuray Özkahraman, Nilüfer Bölükbaşı Balcıoğlu, Merva Soluk Tekkesin, Yusuf Altundağ, Serdar Yalçın

**Affiliations:** 1 Department of Oral Implantology, Faculty of Dentistry, Istanbul University, Istanbul 34093, Turkey; niluferbalcioglu@gmail.com (N.B.B.); serdar.yalcin@istanbul.edu.tr (S.Y.); 2 Department of Tumor Pathology, Institute of Oncology, Istanbul University, Istanbul 34093, Turkey; msoluk@istanbul.edu.tr; 3 Department of Surgery, Faculty of Veterinary, Istanbul University Cerrahpasa, Istanbul 34098, Turkey; ysf.altundag@hotmail.com

**Keywords:** mineralized dentin graft, bone graft, autogenous bone graft, xenograft, osteogenesis, osteoin-duction, osteoconduction

## Abstract

*Background and Objectives*: Dentin grafts have osteoinductive and osteoconductive properties and are considered as an alternative to autogenous graft. This study evaluates the efficacy of autogenous mineralized dentin graft (AMDG) alone or with xenograft and compares it with those of various graft materials used in the treatment of intraosseous bone defects. *Materials and Methods*: The third incisor teeth of six sheep (2–3 years old) were extracted and AMDG was obtained. Six defects were prepared on each tibia of these six sheep: empty defect (group E); autogenous graft (group A), dentin graft (group D), xenograft (group X), autogenous + xenograft (group A + X) and dentin + xenograft (group D + X). Three sheep in each group were sacrificed in the post-operative 3rd and 6th week and the histologic analyses were performed. *Results*: The D and D + X groups showed histological features similar to the other groups in the 3rd and 6th weeks. No statistically significant difference was found regarding the rates of new bone formation between the D and D + X groups (*p* = 1.0) and the other groups at both time intervals (*p* > 0.05). *Conclusions*: Similar results observed in this study between groups A, D, X, A + X and D + X demonstrate that AMDG can be successfully used in the treatment of intraosseous bone defects. Further experimental and clinical studies are needed to be able to evaluate the effectiveness of dentin grafts in different types of indications.

## 1. Introduction

Different types of graft materials are used in the treatment of alveolar bone defects, socket preservation techniques for maintaining the postextraction socket volume and bone augmentation techniques used before or after implant surgeries. Osteogenesis, osteoinduction and/or osteoconduction mechanisms are used to manipulate the regenerative potentials of bone grafts [1]. Osteogenic graft materials contain viable mesenchymal stem cells, osteoblasts and osteocytes to form bone [2]. Osteoinductive grafting materials induce bone-forming cells via the differentiation of multipotent mesenchymal stem cells of the surrounding host bone tissues [2,3]. Osteoconductive graft materials act as a scaffold for osteoblasts adjacent to the grafted area and provide new bone formation [2]. Autogenous bone graft is accepted as the gold standard because of its ability to create new bone formation with the aforementioned three mechanisms [4]. However, it has its own drawbacks, such as having limited obtainability in intraoral areas, requiring general anesthesia to be obtained from extraoral areas, causing an extra surgical trauma and having short resorption time [5,6]. Allografts, xenografts and alloplasts have been produced to obviate these drawbacks of autogenous grafts. Allografts are biomaterials in cancellous, cortical, or demineralized bone matrix forms that are obtained from cadavers and provide new bone formation via osteoconduction and osteoinduction. Alloplastic graft materials (such as calcium sulfate, tricalcium phosphate, and coralline hydroxyapatite, etc.) are synthetic grafting materials that have osteoconductive properties [7]. Xenografts are biocompatible, slow resorbable, osteoconductive graft materials [8]. A combination of different graft materials are also used to boost the positive impact of graft materials on new bone formation. There are ongoing studies on biocompatible, low-cost biomaterials that will help achieve new bone formation with the attributes of natural bone in the shortest timespan possible and will not cause any morbidity. In recent years, teeth that were extracted due to various reasons have started to be used as bone graft materials with high success rates instead of being treated as clinical waste [9,10,11,12]. This is because teeth and bones have very similar structural properties. Teeth and maxillofacial bones stem from the same neural crest cells [13,14]. The percentage of type-1 collagen and hydroxyapatite in their contents are very similar [15,16]. The transforming growth factor-beta (TGF-ß), BMP’s and insulin growth factor I-II present in dentin have a significant role in bone regeneration [17]. Clinically, dentin grafts can be used in block or particle form as demineralized dentin graft (DDG) and mineralized dentin graft (MDG) with respect to their demineralization rates. DDG was first used in the 1960′s by Yeomas and Urist to produce heterotrophic bone regeneration in tendon and muscle tissue as in vivo [18]. The demineralization process aims to expose non-collagen proteins, the growth factors that are effective in bone regeneration. DDG comes with its own drawbacks. There may be a need for an extra surgery session and extra time for demineralization and the demineralization procedure weakens the bone anchorage [19]. However, MDG has been in use for about 40 years. Sperling et al. observed avulsed teeth that they transplanted to socket to be anchylosed and develop replacement resorption in 5 to 8 years [20]. Following the transplantation, the bone gets tightly connected with root dentin or root cement [21]. When the teeth are ground into particles and placed in the extraction socket, the same tightly bound anchylosed connections develop between the bone and the mineralized dentin graft [22,23]. This makes dentin and cement an alternative graft option as an autogenous mineralized tissue source [22,24]. AMDG can be obtained right after tooth extraction and has a very low risk of causing an immune reaction [25]. It also provides support for alveolar bone and soft tissue due to its very slow reformation and helps protect the esthetic appearance [26,27,28].

This histologic and histomorphometric experimental study aims to evaluate the effectiveness of the use of autogenous mineralized dentin grafts alone or combined with xenografts on intraosseous bone defects to be compared with the use of autogenous graft, xenograft and combination of the two that have frequent use in clinical practice.

## 2. Materials and Methods

### 2.1. Study Design

This experimental study was conducted on six male sheep with an average age of 2–3 years and an average weight of 53.36 ± 2.89 kg. The study protocol was approved by the Animal Experimentation Ethics Committee of the ˙Mehmet Akif Ersoy Experimental Research Development and Education Center (approval no: 2018/21; approval date 16 November 2018). We conducted all procedures according to the international ethical guidelines for the welfare and treatment of experimental animals. This manuscript was prepared according to the guidelines proposed by ARRIVE [29].

### 2.2. Sample Size

In order to determine the sample size power analysis was performed using the G*Power (v3.1.9.2) program. For the new bone formation parameter, a minimum difference of 20% between the two groups was predicted to be clinically significant. The effect size was calculated as *d* = 1.818, and the sample size was determined as n:6 for each group to obtain 80% power at the α = 0.05 level. For the study, a total number of 72 defects (36 defects for the 3rd week group and 36 for the 6th week group) were determined.

### 2.3. Surgical Procedures

**Figure 1:**
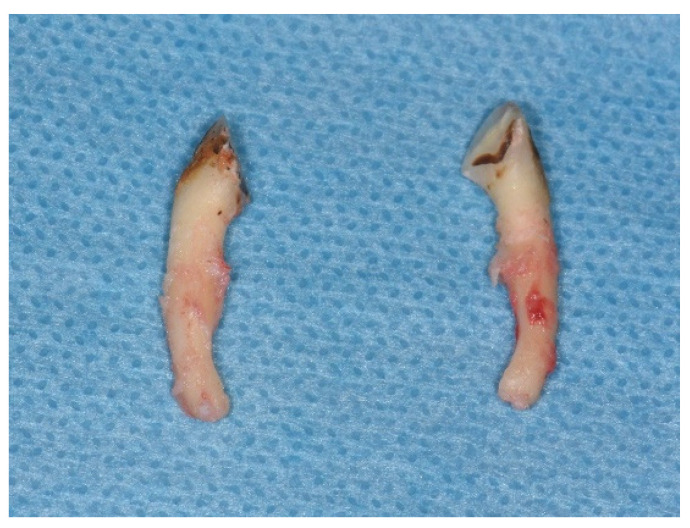
Right and left third incisor teeth on the sheep’s mandibula were extracted.

**Figure 2:**
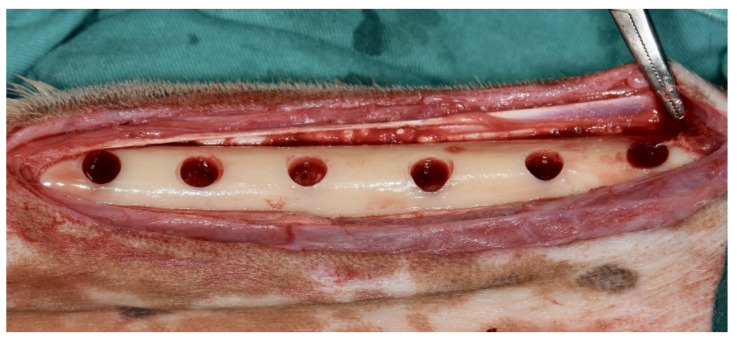
6 defects on each sheep’s tibia were prepared with trephine bur.

**Figure 3:**
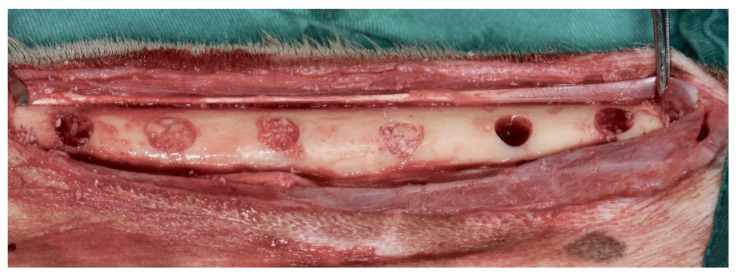
Defects were filled with test group graft materials and control group was left empty.

**Figure 4:**
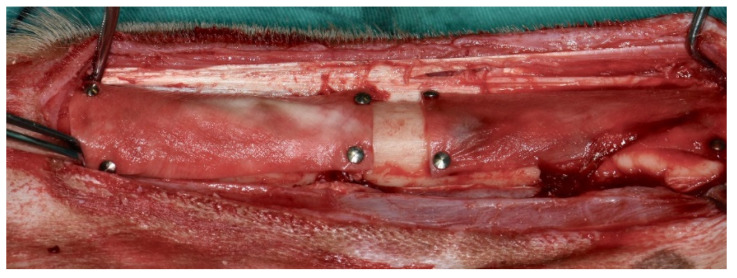
All of the defects surfaces were covered and pinned with collagen membrane.

The animals were fasted 24 h prior to the surgical operation. Following their admission to the operating room, they were intramuscularly administered a combination of atropine sulfate (0.05 mg/kg, Atropin, Vetaş, Istanbul, Turkey), xylazine (0.1–0.2 mg/kg, Bayer, Rompun, Leverkusen, Germany) and ketamine hydrochloride (10–15 mg/kg, Ketalar, Pfizer, New York, NY, USA). Vascular access was established to administer Ringer’s lactate i.v. (Polifarma, Tekirdag, Turkey) at a rate of 5 mL/kg/h after sedation achieved. The animals were transferred to the operating room table, intubated and further anesthetized by administration of 2% isoflurane (Isoflurane^®^, Adeka, Istanbul, Turkey). Intraoral local anesthesia (Ultracain DS forte, Sanofi Aventis, Istanbul, Turkey) was administered then right and left lower third incisor teeth were extracted (Figure 1). The animals were placed in a side-lying position with their legs in question on the bottom. The tibia was shaved from the knee joint to metatarsi. Asepsis and antisepsis of the surgical area were carried out. The medial approach was preferred to reach the shaft of the tibia. An incision of 8–9 cm was performed to the skin from the proximal to the distal of the tibia. The subcutaneous tissues were removed by blunt dissection and the bone’s periosteum was exposed. The periosteum was cut to reach the shaft of the tibia. The full thickness flap was removed and a total of six defects were prepared on each tibia by using a physiodispenser (W&H İmplantmed, Bürmoos, Austria) capable of preparing saline irrigation solution (Isotonic NaCl, Eczacıbaşı-Baxter, Istanbul, Turkey) at 750–800 rpm; 1 cm of space was reserved between the defects that were created using a trephine bur (Kohler Medizintechnik, Stockach, Germany) with 5 mm inner and 6 mm outer diameter (Figure 2). The types of the biomaterials to be applied to defects were determined with the use of a randomization program (Research Randomizer©, Urbaniak GC,& Plous S. 2013. (Version 4.0) Computer software. http://www.randomizer.org/ accessed on 9 December 2021). M ineralized dentin graft (Group D), xenograft (Group X; Bio-Oss^®^, Geistlich Pharma, Wolhusen, Switzerland), autogenous bone graft (Group A; bones collected from defect area and ground with a bone grinder (Ocean System, Bursa, Turkey)), a mixture of 50% mineralized dentin graft and 50% xenograft (Group D + X) and a mixture of 50% autogenous bone graft and 50% xenograft (group A + X) were applied to the prepared defects. One defect was left unfilled as a control (empty defect group, Group E) (Figure 3). The 30 × 40 cm (30 × 40 cm, Bio-Gide^®^, Geistlich Pharma, Wolhusen, Switzerland) collagen membrane was cut into two and all defects were covered and pinned (PinFix, Sedenta, Istanbul, Turkey) with a collagen membrane. (Figure 4). The periosteum was closed with 4–0 resorbable sutures (Vicryl^®^, Ethicon, Istanbul, Turkey) after graft application. Subcutaneous tissue and skin were closed primarily with number 0 resorbable sutures (Prolene^®^, Ethicon, Istanbul, Turkey).

### 2.4. Preparation of Mineralized Dentin Graft

**Figure 5:**
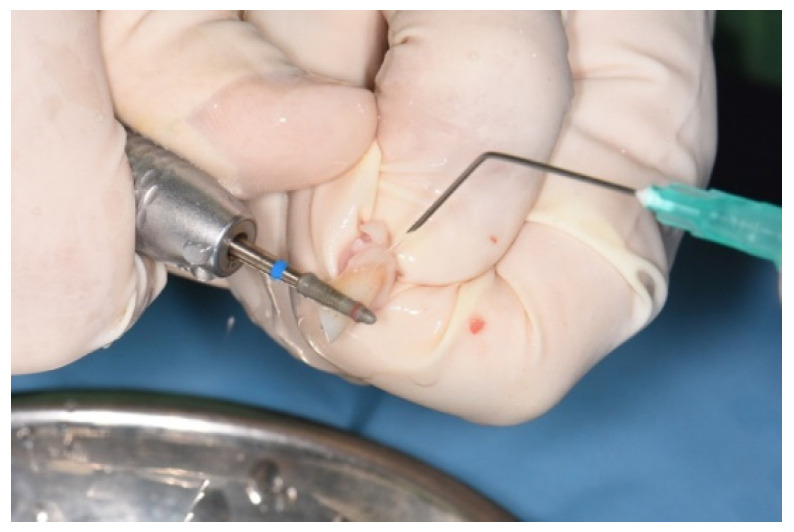
The enamel, cement and periodontal ligaments of the extracted teeth were removed.

**Figure 6:**
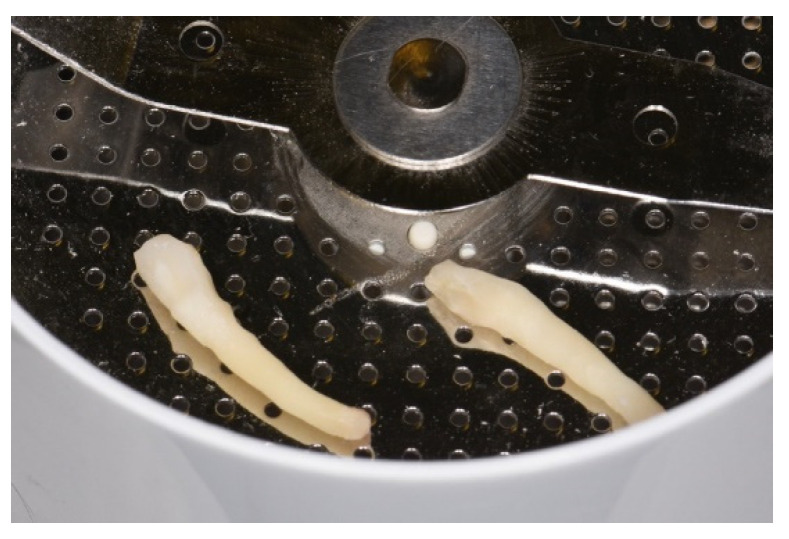
Grinding process of teeth with Kometabio Smart Dentin Grinder.

**Figure 7:**
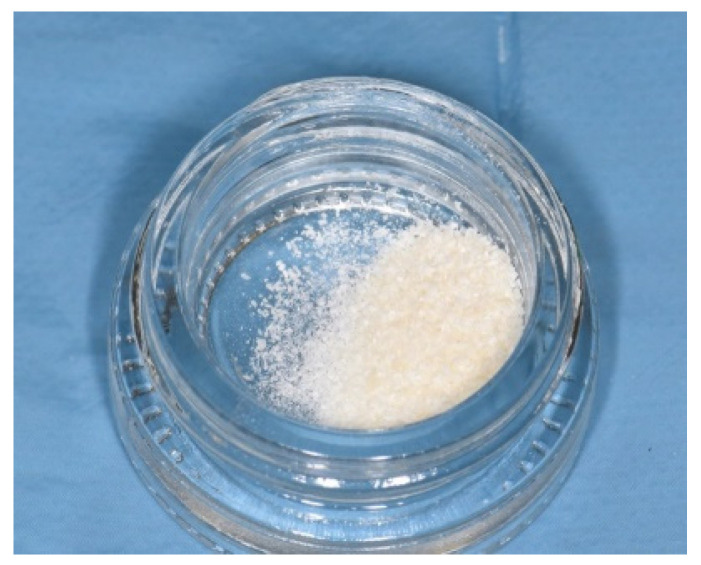
The obtained mineralized dentin graft particles.

The enamel and cement layers of the extracted teeth were removed with a dental handpiece (Figure 5). The teeth were ground with a dentin grinder (Kometabio Smart Dentin Grinder, New York, NY, USA) into particles sized between 300 micron to 1200 micron (Figure 6). The particles were kept in dentin cleanser solution (Kometabio, New York, NY, USA) (20% ethanol + 80% sodium hypochlorite) for 10 min. The solution was then absorbed and removed with a gauze pad. The dentin particles were then kept in phosphate buffered saline (PBS) solution for 3 min to remove the cleanser agent that was used for sterilization. After 3 min, the excessive solution was absorbed with the help of a gauze pad to make the mineralized dentin graft ready for use (Figure 7).

### 2.5. Postoperative Care

Tibias were stabilized with Robert Jones bandage against the risk of fracture at the end of the surgeries. On the 4th day after the surgery, the Robert Jones bandage was removed and the hypochlorous acid (Crystalin, NPH, Turkey) was applied topically to the wound area twice a day for 5 days.30 min before coming out of anesthesia and for 5 days after the operation, the sheep were administered an intramuscular injection of 0.5 mg/kg analgesic meloxicam (Melox 15 mg/1.5 mL Ampul, Nobel, Istanbul, Turkey). A dosage of 20 mg/kg ceftriaxone antibiotic (Novosef 500 mg i.m. Flakon, Zentiva, Istanbul, Turkey) per day was injected into the animals for 5 days to prevent postoperative infection. During the recovery period, the experimental animals were kept in cages separately and their care and general health check were carried out routinely. The cleaning of the living space and the feed and water needs of the animals were provided by the service staff of Mehmet Akif Ersoy Experimental Research Development and Education Center.

### 2.6. Sacrification

The animals were sacrificed randomly on the third and sixth week of the experiment, three of them at a time. A dosage of 50 mg/kg of 10% sodium pentothal solution was used to intravenously euthanize the animals after they were administered xylazine and ketamine to be anesthetized. Following the removal of the soft tissues with dissection, the remaining hard tissues were placed into 10% buffered formalin.

### 2.7. Histologic and Histomorphometric Evaluations

Histologic and histomorphometric evaluations were conducted with single blinded histologist (M.S.T.). The defects were fixed in 10% buffered formalin for a week and then decalcified in a solution of one part of 50% formic acid and one part of 20% sodium citrate. Sagittal sections of defect areas from decalcified pieces were embedded into paraffin blocks. Sections of 5–7 μ were extracted, stained with hematoxylin-eosin (H&E) and examined under light microscope 10× and 20× magnification (Olympus BX60, Tokyo, Japan). Histomorphometric evaluations of new bone formation and residual graft materials were also performed. Images of the chosen areas of each defect were taken at 10× under the same microscope and then computerized. These operations were performed by a camera (Olympus E-330, Olympus Corporation, Tokyo, Japan) connected to the microscope and a computer. The images were processed by the ‘Olympus Soft Imaging System Analysis Five’ (Olympus Corporation) software on the computer to measure the area occupied by bone regeneration and residual graft, and the measurements were compared with the total area. Necrosis and foreign body reaction were graded as 0:no sign, +: existing. Inflammation was scored as 0 (absent), 1 (mild), 2 (moderate) and 3 (severe).

### 2.8. Statistical Analysis

NCSS (Number Cruncher Statistical System) 2007 (Khfsville, UT, USA) software was used for statistical analyses. Descriptive statistical methods (average, standard deviation, median, first quarter, third quarter, frequency, percentage, minimum, maximum) were used to evaluate the results of the study. Shapiro–Wilk test and graphical examinations were used to measure the consistency of the means of quantitative data with normal distribution. Mann–Whitney U test was used for the comparison between two groups of quantitative variables with abnormal distribution and Kruskal–Wallis and Dunn–Bonferroni tests were used for comparisons between groups more than two. For qualitative data comparison, Pearson’s chi-squared and Fisher–Freeman–Halton exact tests were used. Statistical significance was considered to be *p* < 0.05.

## 3. Results

### 3.1. Clinical Findings

No signs of infection, fracture, or wound site opening were observed in the experimental animals. Three experimental animals were sacrificed on the 3rd week and the other three on the 6th week. Histologic and histomorphometric examinations on a total of 72 defects were conducted.

### 3.2. Histologic and Histomorphometric Findings

No signs of necrosis or foreign body reaction were observed in any groups on the 3rd and 6th weeks.

Inflammation was observed on the 3rd week in two defects in group D and one defect in each other; while it was present in one defect in each group on the 6th week. There was no difference between the groups in inflammation scores on the 3rd and 6th weeks (*p* = 0.999 on the 3rd and 6th weeks).

**Figure 8:**
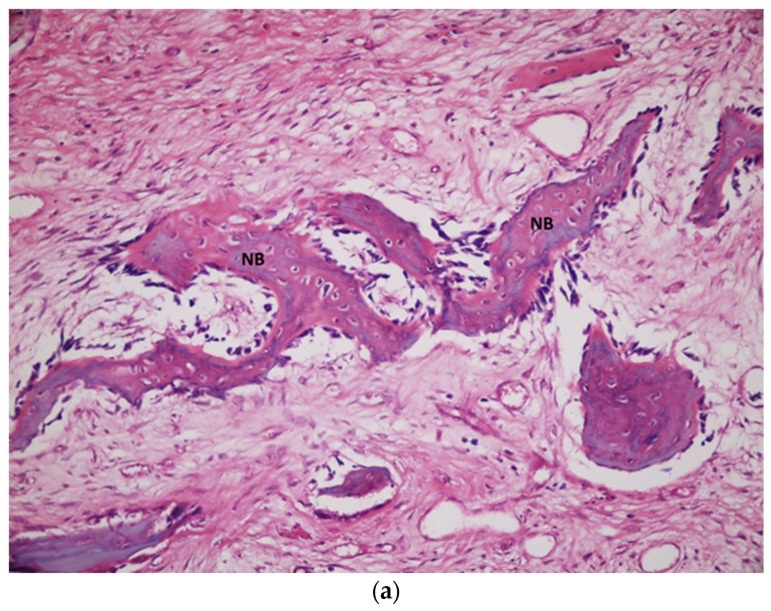

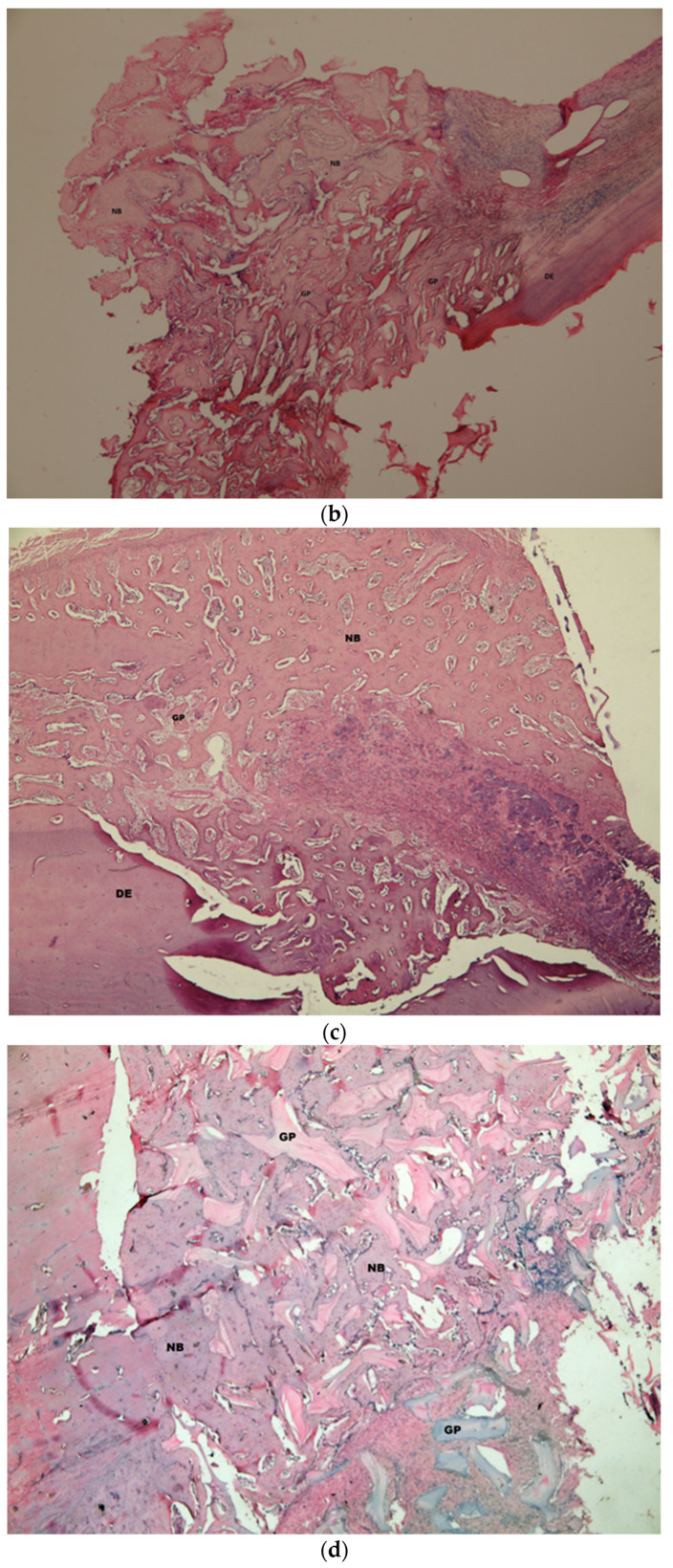

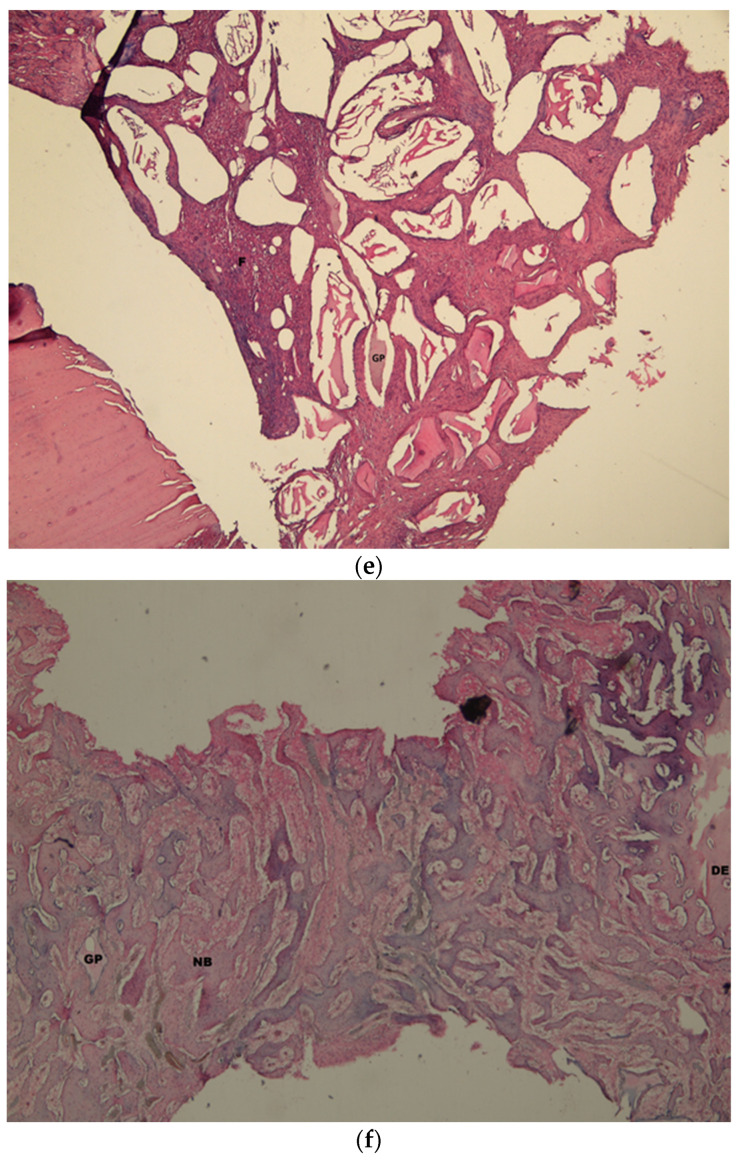
(**a**). Histological view of the empty defect group (group E) on the 3rd week, New bone formation (NB) was observed in the active connective tissue. H&E ×100. (**b**). Histological view of the autogenous group (group A) on the 3rd week. New bone formation (NB) and graft particles (GP) were seen starting from defect edge (ED), H&E ×100. (**c**). Histological view of the dentin group (group D) on the 3rd week. New bone formation (NB) and graft particles (GP) were observed near the defect edge (DE) H&E ×100. (**d**). Histological view of the xenograft group (group X) on the 3rd week. Large new bone formation (NB) and graft particle were determined in the middle of the defect. H&E ×100. (**e**). Histological view of the autogenous + xenograft group (group A + X) on the 3rd week. Mixed fibrosis (F) and graft particles (GP) were seen just near the defect edge (DE) H&E ×100. (**f**). Histological view of the dentin + xenograft group (group D + X) on the 3rd week. New bone formation (NB) and graft particles (GP) were observed between the defect edges (DE) H&E ×100.

Table 1 presents the new bone formation rates measured on the 3rd and 6th weeks. New bone formation on the edges of defects was observed in all but empty defect group (group E) on the 3rd week (Figure 8a–f). In the 3rd week, the highest new bone formation rate was found to be in group A (52.27 ± 3.61), followed by group A + X (48.33 ± 2.53). Similar new bone formation rates were observed in all groups except group E with no statistically significant difference. The value for the group E was measured to be statistically lower than those of groups A, D, X, A + X and D + X (*p* = 0.001; *p* = 0.015, *p* = 0.033; *p* = 0.001; *p* = 0.043; *p* < 0.05, respectively).

**Figure 9:**
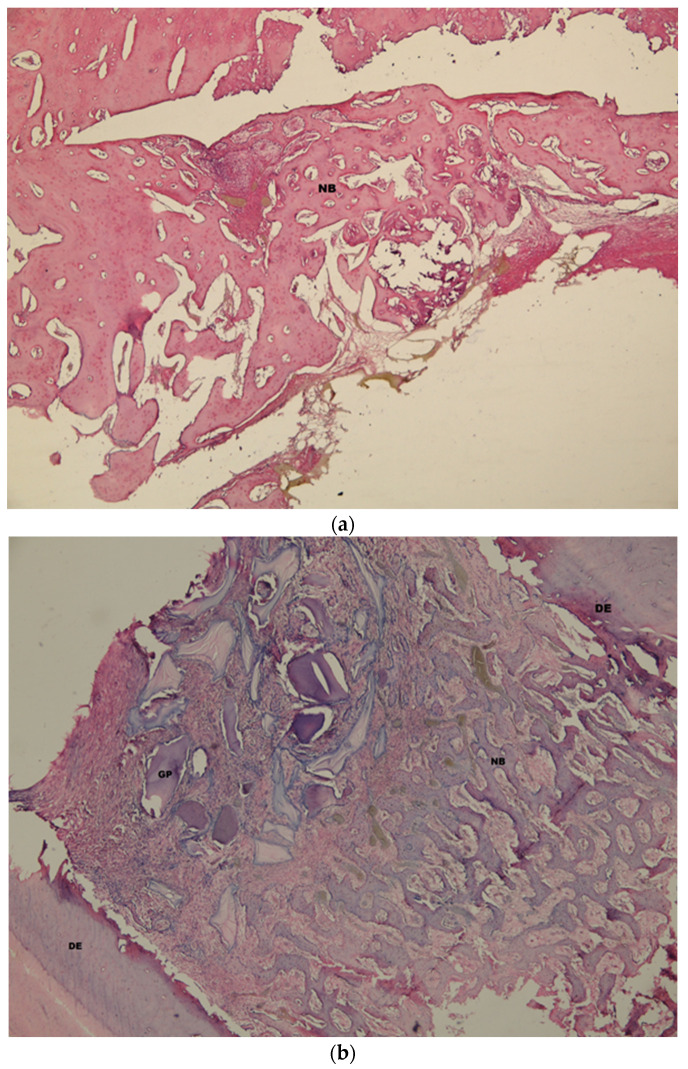

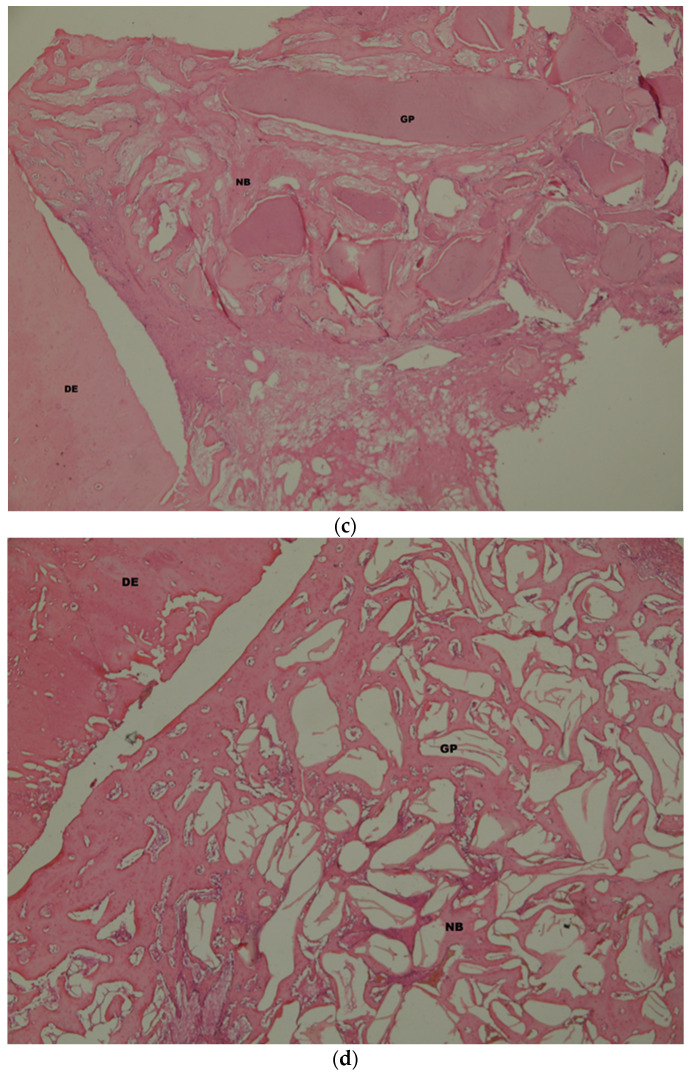

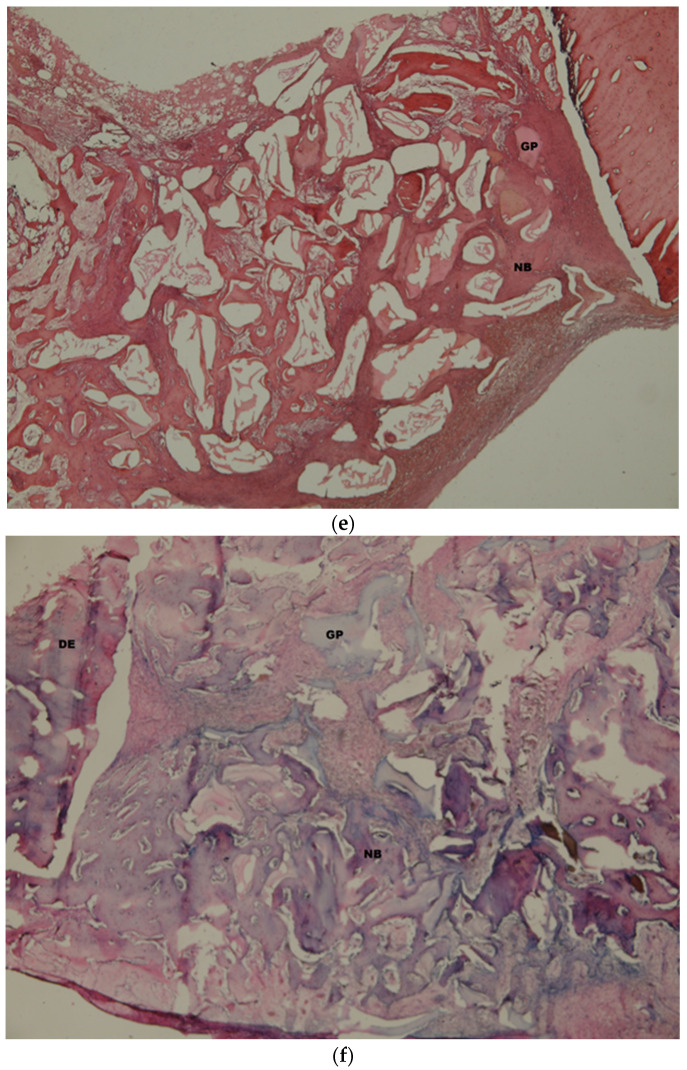
(**a**). Histological view of the empty defect group on the 6th week. Large new bone formation (NB) was determined near the defect edge (DE) H&E ×100. (**b**). Histological view of the autogenous group (group A) on the 6th week. new bone formation (NB) and graft particles (GP) between defect margins (DE). H&E ×100. (**c**). Histological view of the dentin group (group D) on the 6th week. New bone formation (NB) and graft particles (GF) alongside the defect edge (DE) H&E ×100. (**d**). Histological view of the xenograft group (group X) on the 6th week. New bone formation (NB) and graft particles (GF) near the defect edge (DE) H&E ×100. (**e**). Histological view of the autogenous + xenograft group (group A + X) on the 6th week. New bone formation (NB) starting from the defect edges (DE) and rest graft particles (GP) H&E ×100. (**f**). Histological view of the dentin + xenograft group (group D + X) on the 6th week, H&E ×100.

Increased new bone formation areas from the edges to the midsection of defects were observed on the 6th week (Figure 9a–f). The amount of graft particles was decreased and the particles were visible between new bone formation areas. Similar new bone formation rates were measured in groups D, X, A + X, D + X on the 6th week. Group E was observed to have the lowest rate of new bone formation (37.76 ± 1.99) which was statistically significantly lower than those of groups D (69.48 ± 3.29), X (70.23 ± 1.84), A + X (72.11 ± 2.51) and D + X (71.25 ± 3.16) (*p* = 0.030; *p* = 0.045; *p* = 0.024; *p* = 0.001; *p* = 0.001), respectively). Similarly, group A was measured to have statistically significantly lower rates than that of group A + X (*p* = 0.030; *p* < 0.05). There was no statistically significant difference between other groups (*p* > 0.05).

The evaluation of the amount of residual graft in groups D, X, A + X and D + X (Table 2).

The lowest amount of residual graft on the 3rd week was observed to be statistically significantly lower in group A + X (15.83 ± 1.23) than in group X (*p* < 0.001). There was no statistically significant difference between the other residual graft amounts on the 3rd week. There was a decrease in the amount of residual graft on the 6th week compared to the 3rd week. Group X was observed to have higher rates than those of groups D and X + A (*p* = 0.002, *p* = 0.030, respectively).

## 4. Discussion

This study evaluates the histological and histomorphometric efficacy of autogenous mineralized dentin graft alone or mixed with xenograft and compares it with those of various graft materials used for the treatment of intraosseous bone defects. The results were compared with those of the defects that were filled with autogenous graft, xenograft or both which are frequently used in clinical routine. No statistically significant difference was found between the groups where mineralized dentin graft was used alone (group D) or with xenografts (D + X) and the groups of autogenous graft (A), xenograft (X) and autogenous + xenograft (A + X) in the evaluations made on the 3rd and 6th weeks. The new bone formation ratio in the empty control group (E) was found to be statistically significantly lower than other groups in both time periods. To the best of our knowledge, this is the first study ever made in which mineralized dentin graft, autogenous graft, xenograft and their combinations were all compared in the same experimental model. Despite having high osteoinductive properties, the autogenous grafts are frequently used in combination with other grafts in large defects due to being clinically laborious to obtain, having limited obtainability and relatively short resorption time [30]. It is reported that autogenous dentin grafts can be used in many indications because of the structural similarities between autogenous dentin and alveolar bone [31,32]. In a study published in 2018 by Calvo Guirado et al. [33], a histological and histomorphometric evaluation of the extraction sockets of bilateral premolar teeth of dogs was made for the empty control group and AMDG test group after 30 and 90 days. The extracted teeth were ground using Kometabio Smart Dentin Grinder to create 300 µm–1200 µm particles, optimal in size for osteogenity, which were then kept in dentin cleanser solution for 15 min and washed with sterile saline solution. On day 30, a statistically significant difference in new bone formation was observed between the test group (%72.35 ± 0.98) and the control group (%55.87 ± 0.32) (*p* < 0.005), whereas it was %77.18 ± 0.76 for the test group and %58.92 ± 0.32 for the control group on day 90, which is also statistically significant (*p* < 0.05). Similar results were achieved in our study. The new bone formation ratios of the dentin grafts in both time periods were found to be statistically significantly higher than the empty defect group. Mineralized dentin grafts are reported to be usable for socket protection techniques in oral implantology, intraosseous bone defect treatment and sinus lifting operations [19,34,35]. Pohl et al. used AMDG obtained via grinding only from an impacted third molar in sinus lifting operations of 6 patients in 2016. Microscopic examination revealed new bone formation around graft particles due to dentin’s osteoconductive properties [34]. In a study published in 2017, Silvio Valtec et al. [35] removed the enamels, cements and pulps of four patients’ extracted teeth, ground them into particles, mixed the particles with blood and used the obtained grafts for socket protection. After 4 months, they obtained samples from the area with trephine bur and placed an implant into the site. Histological examinations in their study revealed that dentin graft supports bone regeneration and that no inflammation was observed in the graft site. The comparison of bone volumes before the extraction and 1 year after placement of implant revealed 0.76 mm vertical and 1.1 mm horizontal loss. Itzhak Binderman et al. [19] published a study evaluating the effects of the use of mineralized dentin graft particles obtained with Kometabio Smart Dentin Grinder in a bone defect in a periodontally damaged tooth, in immediate implant placement into extraction socket and in sinus lifting surgery. The periodontally damaged root was observed to have fully recovered in 4 months with a 1–2 mm pocket depth and the mineralized dentin applied to the extraction socket in the mandible was observed to have formed new bone in 2 months and then the implants were placed. At the end of a 1-year follow-up period, full recovery was achieved with no issues. The histological and histomorphometric evaluation revealed that dense new bone had formed in the maxilla in the oroantral fistula 3 months after it was filled with mineralized dentin particles. It was noted that the use of mineralized dentin graft resulted in high quality hard bone formation and provided stronger support than demineralized dentin. In their study published in 2011 [36], Chang and Lee filled six patients’ extraction sockets with autogenous mineralized dentin graft and left the other six patients’ sockets empty, and while placing the implants after 3.5 months they conducted a biopsy for histological and histomorphometric evaluation. The mineralized dentin graft sized 200–1000 um was sterilized with ethylene oxide following dehydration, defatting and lyophilization and was comprised of enamel, dentin and cement. There was no statistically significant difference in radiographic analysis. Histomorphometric analysis revealed that the amount of new bone formation in the dentin graft group was %39 ± 7.07 and %20 ± 12.25 in the empty control group and that mineralized dentin statistically increases the amount of new bone formation. In all of these studies, there was either no control group or only empty defect groups were used as control groups. Our study, similar to the aforementioned one, revealed that the use of dentin grafts results in higher new bone formation ratios on the 3rd and 6th weeks compared with empty defect groups.

Dentin grafts can be prepared mineralized or demineralized. Demineralization reveals collagen matrix and growth factors get released, resulting in an increase in regenerative capacity [12,37]. However, the demineralization process leads to the destruction of growth factors and weak osteoconduction [15]. Mineralized dentin graft contains both organic and inorganic materials. It can be prepared and applied in the same clinic session by a dental surgeon following the tooth extraction. Only those grafts that are obtained from autogenous teeth can be used due to the possible risk of infection. In their study, Elfana et al. [38] compared the efficacy of mineralized and demineralized dentin grafts by applying mineralized and demineralized autogenous dentin grafts into extraction sockets and then covering the graft surfaces with a collagen membrane. Histomorphometric and radiologic evaluations were performed in the post-operative 6th month. Radiological examination revealed no statistically significant difference with regard to a decrease in the width of the alveolar bone, the height of the buccal surface of the alveolar bone and lingual height of the alveolar bone between the two groups. In this study, 300 µm–1200 µm mineralized dentin graft particles were prepared with Kometabio Smart Dentin Grinder in order to prevent hopeless teeth from becoming clinic waste and to benefit from the advantages which can be gained only when autogenous grafts are used. In a study in 2016, Pang et al. [39] performed augmentation with a demineralized dentin graft (AutoBt) and xenograft (Bio-Oss^®^) 2–4 weeks after tooth extraction. No statistically significant difference was found with regard to new bone formation between the two groups in the postop 6 months (*p* = 0.606, AutoBt 31.24 ± 13.87, Bio-Oss^®^ %35.00 ± 19.33). The residual graft was found to be %8.95 ± 6.15 in AutoBt and %17.08 ± 16.57 in Bio-Oss (*p* = 2.45). In our study, we achieved similar values for the new bone formation ratios of xenograft and dentin graft, and for residual graft amounts as well.

This experimental study was planned to be performed on sheep to compare different types of graft materials on the same model. Because working on sheep has advantages, such as having bone tissue and remodeling similar to those of humans, being frequently used in bone healing studies because of the possibility to prepare numerous relatively large defects on the same animal and being suitable for working on large defects [40,41]. While evaluating the results of this experimental study, it should be kept in mind that although the organic, inorganic and elemental contents of human tooth dentin and sheep tooth dentin are similar, there are morphological, chemical composition and structural differences between them [42].

The age and gender of the experimental animal may affect bone metabolism. Therefore, young (2–3 years old) sheep were included in the study. In order to act in compliance with ethical rules and not to harm any experimental animals, the right and left lower third incisor teeth of the sheep were extracted. To prevent any fractures in animals’ tibias and because only two teeth were extracted, defects with 6 mm diameter were prepared to obtain dentin graft with enough volume. Our study reveals that defect diameters were appropriately determined because the least amount of new bone formation was observed in empty defects. Two time periods were determined in our study to be able to evaluate the early and late term healing. Due to shorter recovery period in sheep, sacrifices were performed on 3rd and 6th weeks. Studies with long-term follow-up that will evaluate the long-term change in graft volume will prove useful. In implantology, dental implant operations are performed in conjunction with or after the augmentation operations. This study aimed to evaluate the efficacy of mineralized dental graft, therefore, dental implants were not applied to graft areas. In future studies, it will be useful to apply different graft combinations to defects and following a proper healing period to apply dental implants to evaluate bone-implant contact.

## 5. Conclusions

The results of this experimental study conducted on the sheep reveal that the success of autogenous mineralized dentin grafts in the treatment of intraosseous defects is similar to that of autogenous bone, xenograft, autogenous + xenograft and dentin + xenograft. Further experimental and clinical studies are needed to be able to evaluate the effectiveness of dentin grafts in different types of indications.

## Figures and Tables

**Table 1:** The evaluation of the amount of new bone formation by groups.

	The Amount of New Bone Formation (%)			
	3rd Week		6th Week	
	Median (Q1–Q3)	Avg ± SD	Median (Q1–Q3)	Avg ± SD
E	24.4 (23.6–24.8)	24.21 ± 0.87	37.1 (36.4–39.3)	37.76 ± 1.99
A	51 (50.2–55)	52.27 ± 3.61	65 (63.7–66.9)	61.47 ± 1.15
D	45.3 (43–49.3)	46.04 ± 3.36	69.6 (66–72.6)	69.48 ± 3.29
X	45.7 (43.8–47)	45.49 ± 1.77	70 (68.7–71.7)	70.23 ± 1.84
A + X	48.4 (46.5–50.8)	48.33 ± 2.53	71.1 (70.4–74.8)	72.11 ± 2.51
D + X	45.5 (43.6–47.1)	45.37 ± 1.85	70.7 (68.4–74.4)	71.25 ± 3.16
^d^ *p*	<0.001 **		<0.001 **	
**^dd^ *Post Hoc Dunn test***				
E−A	0.001 **		0.030 *	
E−D	0.015 *		0.045 *	
E−X	0.033 *		0.024 *	
E−A + X	0.001 **		0.001 **	
E−D + X	0.043 *		0.006 **	
A-D	*0.420*		*0.645*	
A−X	*0.195*		*0.450*	
A−A + X	*1.000*		0.030 *	
A−D + X	*0.135*		*0.150*	
D−X	*1.000*		*1.000*	
D−A + X	*1.000*		*1.000*	
D−D + X	*1.000*		*1.000*	
X−A + X	*1.000*		*1.000*	
X−D + X	*1.000*		*1.000*	
A + X−D + X	*1.000*		*1.000*	

^d^ Kruskal-Wallis test. ^dd^ Dunn test. * *p* <0.05, ** *p* < 0.01.

**Table 2:** The evaluation of the amount of residual graft material by groups.

	The Amount of Residual Graft Material (%)			
	3rd Week		6th Week	
	Min–Max (Median)	Avg ± SD	Min–Max (Median)	Avg ± SD
D	19.4–24.3 (21)	21.3 ± 1.88	6.4–9.8 (7.4)	7.79 ± 1.29
X	33.8–38.8 (35.5)	35.74 ± 1.93	16.3–24.2 (22.6)	21.57 ± 3
A + X	14–17.2 (16.2)	15.83 ± 1.23	6.5–11.5 (10.1)	9.32 ± 2.07
D + X	22.7–28 (23.7)	24.63 ± 2.26	14.3–19.7 (16.6)	16.97 ± 2.28
^d^ *p*	<0.001 **		<0.001 **	
**^dd^ *Post Hoc Dunn test***				
D−X	*0.075*		0.002 **	
D−A + X	*1.000*		*1.000*	
D−D + X	*1.000*		*0.090*	
X−A + X	0.001 **		0.030 *	
X−D + X	*1.000*		*1.000*	
A + X−D + X	*0.075*		*0.675*	

^d^ Kruskal-Wallis test. ^dd^ Dunn test. * *p* < 0.05. ** *p* < 0.01.
